# AnfO controls fidelity of nitrogenase FeFe protein maturation by preventing misincorporation of FeV‐cofactor

**DOI:** 10.1111/mmi.14890

**Published:** 2022-03-09

**Authors:** Ana Pérez‐González, Emilio Jimenez‐Vicente, Alvaro Salinero‐Lanzarote, Derek F. Harris, Lance C. Seefeldt, Dennis R. Dean

**Affiliations:** ^1^ Department of Biochemistry Virginia Tech Blacksburg Virginia USA; ^2^ Centro de Biotecnología y Genómica de Plantas Universidad Politécnica de Madrid, Instituto Nacional de Investigación y Tecnología Agraria y Alimentaria Madrid Spain; ^3^ Department of Chemistry and Biochemistry Utah State University Logan Utah USA

**Keywords:** *Azotobacter vinelandii*, FeFe protein, FeV‐cofactor, metalloprotein, nitrogen fixation, nitrogenase

## Abstract

*Azotobacter vinelandii* produces three genetically distinct, but structurally and mechanistically similar nitrogenase isozymes designated as Mo‐dependent, V‐dependent, or Fe‐only based on the heterometal contained within their associated active site cofactors. These catalytic cofactors, which provide the site for N_2_ binding and reduction, are, respectively, designated as FeMo‐cofactor, FeV‐cofactor, and FeFe‐cofactor. Fe‐only nitrogenase is a poor catalyst for N_2_ fixation, when compared to the Mo‐dependent and V‐dependent nitrogenases and is only produced when neither Mo nor V is available. Under conditions favoring the production of Fe‐only nitrogenase a gene product designated AnfO preserves the fidelity of Fe‐only nitrogenase by preventing the misincorporation of FeV‐cofactor, which results in the accumulation of a hybrid enzyme that cannot reduce N_2_. These results are interpreted to indicate that AnfO controls the fidelity of Fe‐only nitrogenase maturation during the physiological transition from conditions that favor V‐dependent nitrogenase utilization to Fe‐only nitrogenase utilization to support diazotrophic growth.

## Graphical abstract

*Azotobacter vinelandii* produces three nitrogenase isozymes designated Mo‐dependent, V‐dependent, and Fe‐only on the basis of the heterometal contained in their corresponding active site cofactors. Inactivation of *anfO* results in misincorporation of the active site cofactor associated with V‐dependent nitrogenase into the Fe‐only nitrogenase. This observation indicates that AnfO preserves the fidelity of Fe‐only nitrogenase by preventing incorrect active site cofactor insertion during the transition phase to conditions that favor Fe‐only nitrogenase utilization.

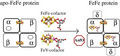

## 1 INTRODUCTION

**FIGURE 1:**
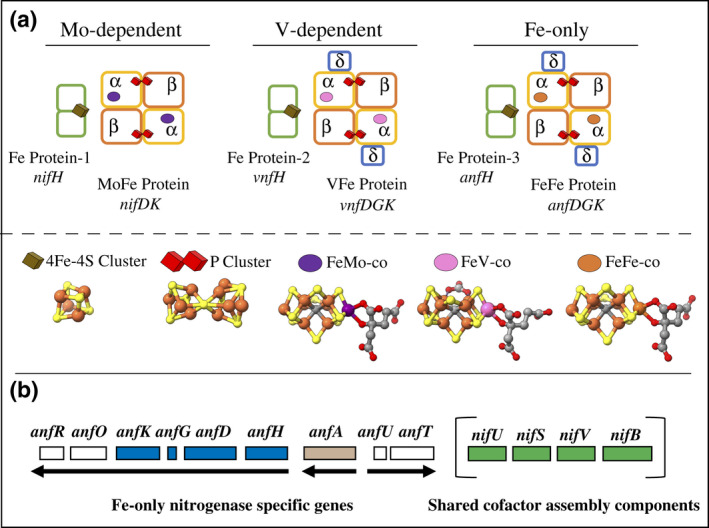
Schematic representation of the catalytic components of the three nitrogenases found in *Azotobacter vinelandii*. (a) the MoFe protein is a tetramer that contains two pairs of metalloclusters: P‐cluster located at the interface of the α‐β subunits, and FeMo‐cofactor located within the α‐subunits. VFe‐ and the FeFe‐nitrogenase architectures are like the Mo‐nitrogenase, except for the presence of a third subunit, δ, encoded by *vnfG* and *anfG* genes, respectively. Protein designations and their encoding genes are indicated below each component. The corresponding Fe proteins for the three systems are indicated as 1, 2 or 3. Atoms in the structures are indicated as follows: yellow, sulfur; gray: carbon; red: oxygen; orange: iron; purple, molybdenum; pink, vanadium. The coordinates for the different clusters represented were extracted from the following PDB files: [4Fe‐4S] cluster from PDB 2NIP; P‐cluster from PDB 3U7Q; FeMo‐cofactor and FeV‐cofactor from PDB 3U7Q and PDB 5N6Y, respectively. FeFe‐cofactor has been inferred from FeMo‐cofactor with Fe replacing Mo although its structure has not yet been established by crystallographic methods. Structures were visualized with ChimeraX software (Pettersen et al., 2020). (b) Schematic representation of the genes associated with the Fe‐only nitrogenase in *A. vinelandii*. Four *nif* gene products are necessary for the maturation of the FeFe protein (bracketed, in green). Nine genes are specifically associated with the Fe‐only system, designated as *anf*. The genes encoding the catalytic components (*anfHDGK*) are colored in blue; *anfA*, which encodes for the transcriptional activator, is colored in brown

Biological nitrogen fixation, the nucleotide‐dependent reduction of N_2_ to ammonia, is catalyzed by the nitrogenases for which three structurally and functionally similar but genetically distinct types have been identified (Bishop & Joerger, 1990; Harris et al., 2019; Joerger & Bishop, 1988). One differentiating feature among the nitrogenases is the presence of an apical heterometal (Mo, V, or Fe) contained within their corresponding catalytic cofactors comprised of an Fe‐S‐C core (Figure 1). Accordingly, they have been, respectively, designated as Mo‐dependent, V‐dependent, or Fe‐only nitrogenases (Eady, 1996). Mo‐dependent nitrogenase is a more efficient N_2_ reduction catalyst than V‐dependent nitrogenase, which, in turn, is more efficient than Fe‐only nitrogenase (Harris et al., 2019; Harris, Yang, et al., 2018). Catalytic N_2_ reduction efficiency refers to the relative allocation of electron to N_2_ reduction relative to non‐productive proton reduction. In the case of *Azotobacter vinelandii*, whose genome encodes all three nitrogenases (Setubal et al., 2009), there is selective expression of a particular nitrogenase isozyme depending upon the availability of Mo or V in the growth medium. For example, when *A. vinelandii* is cultured under conditions that demand N_2_ reduction for growth, the expression of Mo‐nitrogenase is favored when Mo is available. However, when *A. vinelandii* is cultured in the absence of Mo, but in the presence of V, expression of V‐nitrogenase is favored and, in the absence of either Mo or V, the Fe‐only nitrogenase is expressed (Luque & Pau, 1991).

Catalytic units of the nitrogenases are comprised of two‐component proteins. For the Mo‐dependent nitrogenase, these components include Fe protein‐1, which supplies electrons to the other component, designated MoFe‐protein, which contains the site for substrate binding and reduction (Bulen & LeComte, 1966; Einsle & Rees, 2020). The catalytic units of the two other systems have analogous components involving an Fe protein‐2 and VFe protein and an Fe protein‐3 and FeFe protein. The catalytic components of the different nitrogenase types, as well as the complex metal‐containing cofactors that provide their respective substrate‐binding and reduction sites, designated FeMo‐cofactor, FeV‐cofactor, and FeFe‐cofactor (Seefeldt et al., 2020), are schematically shown in Figure 1. The precursor to all three catalytic cofactors is an 8Fe‐9S‐C cluster designated NifB‐co. For FeMo‐cofactor and FeV‐cofactor formation, NifB‐co is further processed on molecular scaffolds, respectively designated NifEN and VnfEN, resulting in the incorporation of an organic constituent, homocitrate, and the correct heterometal (Burén et al., 2020). Intact FeMo‐cofactor or FeV‐cofactor is then inserted into an immature species of their respective cognate protein partners designated apo‐MoFe protein or apo‐VFe protein. In contrast, conversion of NifB‐co to FeFe‐cofactor, which might only require incorporation of homocitrate, is completed without the assistance of an assembly scaffold analogous to NifEN or VnfEN (Pérez‐González et al., 2021).

In recent years, there has been a gathering interest in understanding the assembly and catalytic properties of Fe‐only nitrogenase because of its simplicity with respect to the genetic determinants required for its formation, thereby making it a favored target for transferring a capacity for nitrogen fixation to model eukaryotic systems. In *A. vinelandii*, although several gene products (*nifU*, *nifS, nifV*, and *nifB*) are required for maturation of all three nitrogenase types (Bishop & Joerger, 1990; Kennedy & Dean, 1992), there are nine genes (*anfH*, *anfD*, *anfG*, *anfK*, *anfO*, *anfR*, *anfA*, *anfU*, and *anfT*) uniquely associated with the Fe‐only nitrogenase (Joerger et al., 1989; Setubal et al., 2009; Varghese et al., 2019). Among the *anf* genes only those encoding the structural components *anfH* (Fe protein‐3), *anfDGK* (encoding the FeFe protein subunits), *anfA* (positive regulatory element), and *anfO* (function not known) are required to form an active Fe‐only nitrogenase (Joerger et al., 1989; Mylona et al., 1996; Premakumar et al., 1998). Although an *A. vinelandii* strain inactivated for *anfO* was shown to not grow in the absence of a fixed nitrogen source (Mylona et al., 1996), genetic reconstruction experiments using *Escherichia coli* as the host revealed that *anfO* is not necessarily required for heterologous production of an active Fe‐only nitrogenase (Yang et al., 2014). In the present work, a combination of genetic and biochemical analyses were used to reconcile these apparently contradictory observations and reveal that AnfO serves to preserve the fidelity of FeFe protein maturation in *A. vinelandii* by preventing the misincorporation of FeV‐cofactor.

## 2 RESULTS

### 2.1 Experimental rationale and strain construction

Prior work from the laboratory of Paul Bishop established that an *A. vinelandii* strain deleted for *anfO* loses the capacity for Fe‐only nitrogenase‐dependent diazotrophic growth (Mylona et al., 1996). It was also shown that whole cells of the *anfO*‐deletion strain retained a capacity for the reduction of the artificial nitrogenase substrate acetylene but does not reduce the natural substrate N_2_ (Mylona et al., 1996). These features mirror the phenotype of Mo‐dependent nitrogenase produced by a *nifV*‐deficient strain (Hoover et al., 1988; Kennedy & Dean, 1992; Liedtke et al., 2021; McLean & Dixon, 1981). NifV catalyzes the formation of homocitrate, the organic acid constituent common to FeMo‐co, FeV‐cofactor, and FeFe‐cofactor. In the case of Mo‐dependent nitrogenase, inactivation of *nifV* results in the incorporation of citrate as the organic acid constituent of FeMo‐cofactor rather than homocitrate (Liang et al., 1990; Liedtke et al., 2021). Substitution of citrate within FeMo‐cofactor leads to a very slow diazotrophic growth phenotype and production of a MoFe protein that retains a capacity for acetylene reduction but is only a very poor catalyst for N_2_ reduction. Such similarities to the *anfO*‐deficient phenotype in the case of Fe‐only nitrogenase led to speculation that AnfO might be involved either in the modification of FeFe‐cofactor or its polypeptide environment or, perhaps, in the attachment of homocitrate (Mylona et al., 1996). The initial goal of the present work was to explore these possibilities by asking if the previously reported growth phenotype associated with *anfO* inactivation could be reproduced and whether FeFe‐only protein isolated from an AnfO‐deficient strain exhibits altered catalytic properties when compared to FeFe protein produced by the wild‐type strain. To perform these experiments, strains inactivated for genes encoding the MoFe protein, the VFe protein, and either having an intact *anfO* or an in‐frame deletion within *anfO* were constructed. All strains constructed also have a 42,096 base‐pair genomic deletion that includes portions of the Mo regulon and are therefore deficient in Mo acquisition (Noar et al., 2015; Premakumar et al., 1996). Deletion of genes encoding both the MoFe protein and VFe protein prevents their potential to support diazotrophic growth, thereby simplifying phenotypic analyses. Furthermore, inactivation of Mo accumulation prevents Mo‐dependent repression of Fe‐only nitrogenase expression and inactivation of the VFe protein prevents the V‐dependent repression of Fe‐only nitrogenase expression (Luque & Pau, 1991; Premakumar et al., 1992). Consequently, growth and biochemical phenotypes reported here can only be assigned to activities associated with Fe‐only nitrogenase. The genotypes of all strains used in the present work are listed in Table S1.

### 2.2 V is responsible for the null diazotrophic growth phenotype of the *anfO* deletion strain

**FIGURE 2:**
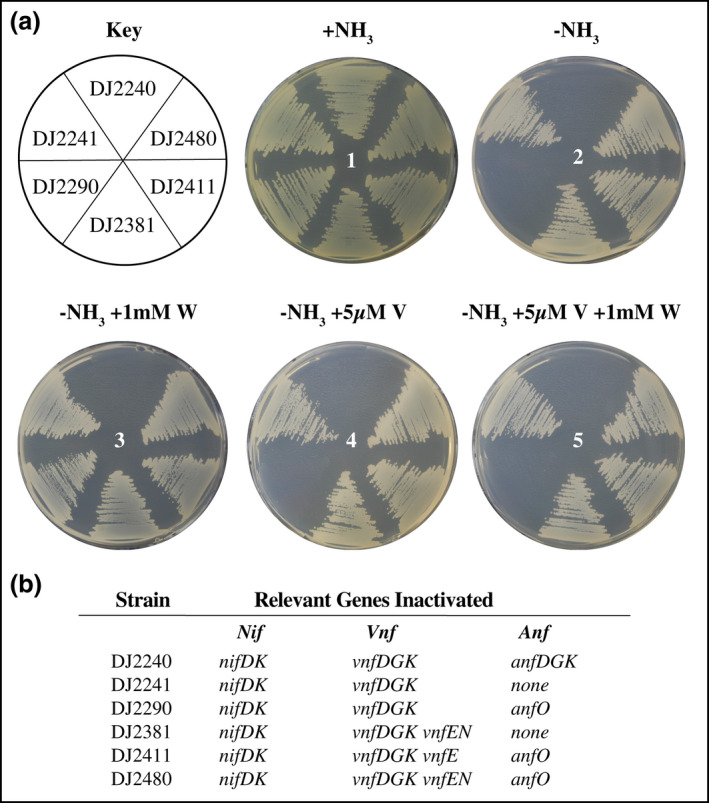
Phenotypic characterization of different *Azotobacter vinelandii* strains containing deletions in *anfO* and/or *vnfEN* genes. (a) Strains expressing FeFe nitrogenase were cultured on Burk’s medium agar plates containing a fixed nitrogen source (+NH_3_) (plate 1) or under different diazotrophic growth conditions (–NH_3_) (plates 2–5). V and W were added to the growth media as indicated for each condition. Strains were cultured on agar plates for 5 days. (b) Relevant genes inactivated for each strain. Refer to Table S1 for a complete genotypic description of the strains indicated. The key observations are the reversal of the incapacity for diazotrophic growth observed for DJ2290 when W is added to the media (plate 3) or when *vnfEN* are inactivated in combination with a deletion in *anfO* (DJ2411 and DJ2480, plates 2, 4 and 5)

The growth capacities of various strains used in the present work are shown in Figure 2. All strains grow when fixed nitrogen, in this case, ammonium acetate, is added to the growth medium (Figure 2, plate 1). However, under conditions that demand N_2_ reduction for growth (no fixed nitrogen source added to the growth medium), a control strain disabled for all three nitrogenase types (DJ2240) is unable to grow (Figure 2, plate 2). Furthermore, a strain deleted for *anfO* (DJ2290) but otherwise having an intact capacity for producing Fe‐only nitrogenase components is also incapable of diazotrophic growth (Figure 2, plate 2). These results agree with those previously reported by Bishop and coworkers (Mylona et al., 1996) but apparently are not in agreement with genetic reconstruction experiments reported by Dixon, Wang, and coworkers (Yang et al., 2014).

To examine the biochemical phenotype associated with the loss of AnfO, FeFe protein was isolated from an *anfO* deletion strain and compared to FeFe protein isolated from cells having an intact *anfO*. For this purpose, large‐scale growth involved culture of the appropriate strain in liquid media to a high density using a fixed nitrogen source followed by expression of Fe‐only nitrogenase by continued incubation once the fixed nitrogen source was exhausted. Surprisingly, the catalytic properties with respect to N_2_ reduction, proton reduction, and reduction of acetylene to either ethylene or ethane were nearly the same for FeFe protein prepared from either genetic background (Table 1). These results agree with those from heterologous genetic reconstruction experiments using *E. coli* reported by Dixon, Wang, and colleagues but are not in agreement with whole‐cell assays using an *anfO* deletion strain reported by Bishop and colleagues.

**TABLE 1:** Catalytic properties and metal content of FeFe proteins isolated from different genetic backgrounds

Purified FeFe protein			Specific activities				Metal content	
FeFe protein source	Genes inactivated	V added to the media	Protons (1 atm Ar)	N_2_ (1 atm) and protons		C_2_H_2_ (0.4 atm)	Fe content	V content
			nmol	nmol	nmol	nmol C_2_H_4_/min/mg	mol Fe/mol	mol V/mol
			H_2_/min/mg	NH_3_/min/mg	H_2_/min/mg	nmol C_2_H_6_/min/mg	FeFe protein	FeFe protein
DJ2241	*nifDK, vnfDGK*	–	773 ± 38	171 ± 1	435 ± 15	196 ± 14 6.0 ± 0.2	26 ± 0^a^	0.07 ± 0.00^b^
DJ2290	*nifDK, vnfDGK, anfO*	–	734 ± 33	172 ± 7	475 ± 20	190 ± 2 6.8 ± 1.3	29 ± 0^a^	0.06 ± 0.00^b^
DJ2241	*nifDK, vnfDGK*	5 μM	520 ± 13	55 ± 4	374 ± 6	97 ± 5 1.6 ± 0.1	28 ± 1	1.05 ± 0.03
DJ2290	*nifDK, vnfDGK, anfO*	5 μM	303 ± 13	0 ± 0	291 ± 7	29 ± 2 0.8 ± 0.1	32 ± 0^a^	1.65 ± 0.00^b^
DJ2381	*nifDK, vnfDGK, vnfEN*	5 μM	903 ± 34	213 ± 10	508 ± 32	236 ± 6 N.D.	31 ± 1	0.12 ± 0.03
DJ2411	*nifDK, vnfDGK, anfO, vnfE*	5 μM	934 ± 43	201 ± 2	465 ± 23	195 ± 16 N.D.	N.D.	N.D.
DJ2480	*nifDK, vnfDGK, anfO, vnfEN*	5 μM	971 ± 33	228 ± 3	510 ± 20	202 ± 21 N.D.	27 ± 0^a^	0.11 ± 0.00^b^

*Notes*: FeFe proteins were purified from cells grown in the presence or absence of 5 μM V as indicated. Metal contents were quantified by ICP‐MS. Molar ratios were calculated based on the molecular weight of the FeFe protein α_2_β_2_δ_2_ complex. Data presented are the average from at least three independent determinations.

Abbreviation: N.D., not determined.

^a^ Standard deviation value <1;

^b^ Standard deviation value <0.001.

We considered the possibility that these apparently contradictory results might be a consequence of the different experimental conditions used to elicit the corresponding growth and biochemical phenotypes. Namely, in the case of the null diazotrophic growth phenotype evident for a strain deleted for *anfO* (Figure 2, plate 2) petri plates were inoculated with cells at a very low density, whereas for biochemical analyses of FeFe protein, cells were grown to a high density prior to inducing Fe‐only nitrogenase expression. It, therefore, seemed possible a metabolite is present in the culture media that results in a null diazotrophic growth phenotype for the *anfO* deletion strain when cultured at low density but is metabolically removed when cells are cultured to high density prior to Fe‐only nitrogenase expression. In other words, the growth of the *anfO* deletion strain to a high density prior to Fe‐only nitrogenase expression might result in the sequestration of an inhibitory metabolite present at low concentration within a relatively small sub‐population of cells. Obvious candidates for such an inhibitory metabolite include Mo and V. Here, we focused on the possibility that a low level of V present in the growth medium is the inhibitory metabolite because strains used in the present work are incapacitated for Mo acquisition and therefore trace levels of Mo are unlikely to account for the observed phenotypes. To assess if trace levels of V might be responsible for the inability of Fe‐only nitrogenase to reduce N_2_ in the case of the *anfO* deletion strain, 1 mM W (WO_4_^−2^) was added to the growth medium because W was anticipated to be an antagonist for both Mo and V accumulation (Keeler & Varner, 1957). Indeed, the addition of W rescues the null diazotrophic growth phenotype of the *anfO* deletion strain. Importantly, rescue of the null diazotrophic growth phenotype of the *anfO* mutant is reversed by the addition of 5 μM V (VO_3_^−1^) to media that is also supplemented with 1 mM W (Figure 2, plate 5). These results pinpoint V as the metabolite responsible for the null diazotrophic growth phenotype of the *anfO* deletion strain. Notably, the V‐dependent null growth phenotype of the *anfO* deletion strain must be associated with a biochemical defect in Fe‐only nitrogenase rather than its expression because V‐dependent repression of Fe‐only nitrogenase has been disabled in the *anfO* deletion strain (Luque & Pau, 1991). In separate experiments, it was also confirmed that a trace level of V is responsible for the inability of an *anfO* deletion strain to sustain diazotrophic growth. This was done by rescue of the null diazotrophic growth phenotype when the *anfO* deletion strain was cultured on petri plates prepared using purified agarose, rather than agar, and trace levels of V removed by chelex treatment of the mineral salts solution used for media preparation (Figure S1, plate 2). When 5 μM V was added to the chelex‐treated media, the null diazotrophic growth phenotype of the *anfO* deletion strain was restored (Figure S1, plate 3). To be certain that the observed null growth phenotype associated with deletion of *anfO* is directly linked to loss of AnfO function a strain was constructed, DJ2622, that has the endogenous *anfO* gene deleted and intact *anfO* separately placed at a different genomic location whose expression is under control of the arabinose regulatory elements from *E. coli* as previously described for similar genetic constructs (Dos Santos et al., 2007). This strain can only grow on Petri plates under diazotrophic conditions if arabinose is added to the growth medium to stimulate *anfO* expression (Figure S2).

Based on the above results, the biochemical consequences on FeFe protein activity resulting from the addition of excess V during large‐scale growth of strains having either an intact *anfO*, or a deletion in *anfO*, were examined (Table 1). This analysis revealed that FeFe protein produced by the *anfO* deletion strain grown in the presence of V exhibits no capacity for N_2_ reduction yet retains substantial proton reduction activity and modest levels of acetylene reduction activities. These results are like those previously reported using whole‐cell assays (Mylona et al., 1996). N_2_ reduction activity is also lower for FeFe protein produced by cells having an intact *anfO* gene when grown in the presence of V, but in this case, there is clearly detectable N_2_ reduction activity and there is no effect on the capacity for diazotrophic growth (Figure 2, plate 4). Metal analysis (Table 1) revealed that FeFe protein purified from both sources contain V at levels higher than FeFe protein prepared from cells that have not been grown in the presence of V, although the V content is more substantial in FeFe protein produced by the *anfO* deletion strain (Table 1). These results indicate that when challenged with a high concentration of V in the growth medium AnfO does not completely prevent incorporation of V into FeFe protein. In a separate analysis, it was found that cofactor extracted from FeFe protein prepared from strain DJ2290, deleted for *anfO*, and grown in the presence of V contains approximately 1 V/10Fe which compares favorably to the ideal ratio of 1 V/7Fe for FeV‐cofactor (Sippel & Einsle, 2017). Thus, it is concluded that FeFe protein that has lost the capacity to reduce N_2_ is a hybrid species that carries a V‐containing catalytic cofactor. Note that the δ subunit is present in all the isolated samples, including the hybrid species containing the V‐catalytic cofactor (Figure S3).

### 2.3 Deletion of *anfO* results in the incorporation of FeV‐cofactor into FeFe protein

There are two possibilities that could explain the high level of V incorporation into FeFe protein produced in the absence of AnfO. One possibility is that, in the absence of AnfO, V becomes adventitiously incorporated into FeFe‐cofactor during its assembly. In this case, because NifB‐co is processed to FeFe‐cofactor without the aid of an intermediate biosynthetic scaffold (Pérez‐González et al., 2021), direct incorporation of V into FeFe‐cofactor would need to occur. The other possibility is that FeV‐cofactor, whose synthesis is separately completed on the VnfEN molecular scaffold (Rüttimann‐Johnson et al., 1999), competes with FeFe‐cofactor for occupancy during FeFe protein maturation. These possibilities were differentiated in two different ways. First, 11 pseudo‐revertant strains that suppress the null diazotrophic growth phenotype of the *anfO* deletion strain (DJ2290) were isolated. All 11 of the suppressor mutations were found to map to the *vnfENX* loci. Two of the suppressor mutations were identified by DNA sequence analysis; in one strain (DJ2411) a single bp frame shift “A” insertion after *vnfE* nucleotide 755 was found, and the other strain (DJ2490) carries a one bp frame shift “G” deletion of *vnfE* nucleotide 827. The diazotrophic growth phenotype of DJ2411 is shown in Figure 2, plate 2. A second complementary approach involved the directed deletion of *vnfE* and *vnfN* in a strain (DJ2480) that is also deleted for *anfO*. The deletion of *vnfEN* resulted in rescue of the null diazotrophic growth phenotype associated with loss of AnfO function (Figure 2, plate 2). Furthermore, FeFe protein isolated from either DJ2411 or DJ2480 grown in the presence of 5 μM V exhibits high levels of N_2_ reduction activity and only basal levels of V incorporation (Table 1). Based on these results, it is concluded that cells cultured in the presence of V and the absence of AnfO incorporate FeV‐cofactor into FeFe protein. It is also noteworthy that the lower activity for N_2_ reduction and significant incorporation of V into FeFe protein produced by a strain having an intact *anfO*, when grown in the presence of 5 μM V, is eliminated when *vnfE* or *vnfEN* are inactivated (Table 1).

### 2.4 The CO reduction capacity of the hybrid FeFe protein‐containing FeV‐cofactor retains the characteristic feature of FeFe protein‐containing FeFe‐cofactor

**FIGURE 3:**
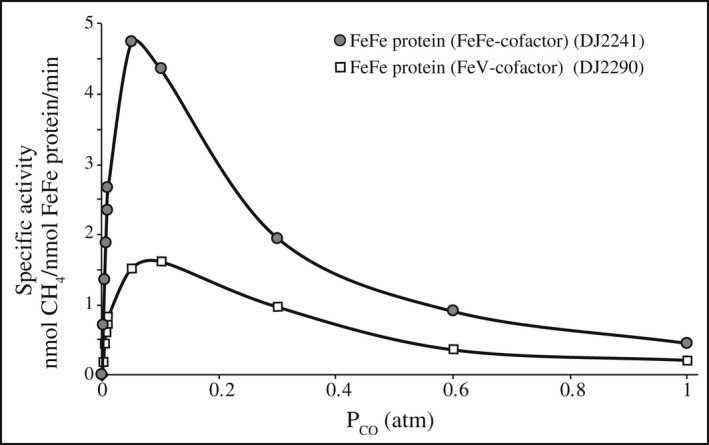
Specific activities for CO reduction by the FeFe protein loaded with FeFe‐cofactor (•) or FeV‐cofactor (□). Specific activities for CH_4_ are shown as a function of partial pressure of CO (P_CO_) in atm. Data are the average of at least two independent experiments. Extremely low traces of C_2_H_4_ were detected in both cases

Both V‐dependent nitrogenase and Fe‐only nitrogenase catalyze the reduction of CO to produce hydrocarbons (Jasniewski et al., 2020). However, the pattern of product formation is quite different for the different systems. In the case of V‐dependent nitrogenase, ethylene is the primary CO reduction product, whereas for Fe‐only nitrogenase, methane is the primary CO‐reduction product (Harris et al., 2020). The ability to produce a hybrid FeFe protein that contains FeV‐cofactor rather than FeFe‐cofactor permitted the determination of whether such differentiating CO‐reduction profiles are inherent to the corresponding cofactor structures, the nature of their polypeptide environments, or some combination of these features. Figure 3 compares the CO reduction products of FeFe protein‐containing FeFe‐cofactor as well the hybrid FeFe protein prepared from the strain deleted for *anfO* that contains FeV‐cofactor. Although the efficiency for methane production is lower for the hybrid FeFe protein, the reduction pattern is the same as FeFe protein‐containing FeFe‐cofactor. Furthermore, unlike VFe protein there is no significant CO reduction by the hybrid FeFe protein to yield short‐chain hydrocarbons. Thus, differentiating features for CO reduction exhibited by V‐dependent nitrogenase and Fe‐only nitrogenase arise from differences in their primary structures rather than the composition of their cognate cofactors.

## 3 DISCUSSION

**FIGURE 4:**
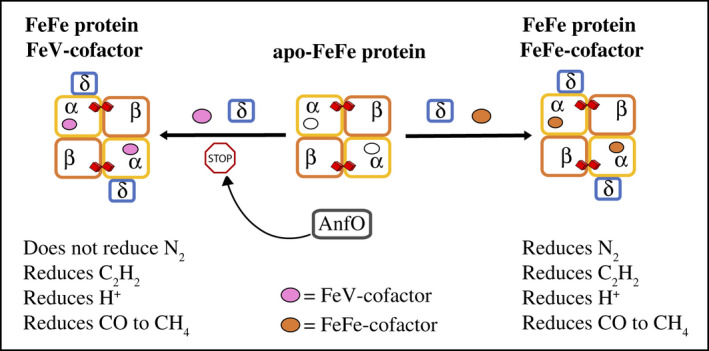
Schematic representation of the proposed role of AnfO. Under normal conditions, AnfO blocks the incorporation of FeV‐cofactor into apo‐FeFe protein thereby ensuring the fidelity of FeFe‐cofactor incorporation (rightward arrow). In the absence of AnfO incorporation of FeV‐cofactor is favored leading to the accumulation of a hybrid species (leftward arrow) that cannot reduce N_2_. Attachment of the δ subunit requires either FeFe‐cofactor (Pérez‐González et al., 2021) or FeV‐cofactor (Yang et al., 2021)

The scheme shown in Figure 4 summarizes the findings presented in the current work. Under conditions where both FeFe protein and FeV‐cofactor are produced the fidelity of FeFe protein is preserved by the action of AnfO which prevents the misincorporation of FeV‐cofactor. The inactivation of *A. vinelandii anfR*, which is located immediately downstream of *anfO* (Figure 1b), also results in poor diazotrophic growth and whole cells that retain some acetylene reduction activity but no apparent N_2_ reduction activity (Mylona et al., 1996). However, owing to the leaky diazotrophic growth phenotype associated with inactivation of *anfR* its possible role in preserving the fidelity of cofactor insertion into FeFe protein was not explored in the present work. Nevertheless, the co‐location of *anfO* and *anfR*, together with phenotypes associated with their inactivation, suggests they are both involved in the same process. Given the nature of the genetic constructs used in the present work, the possibility that AnfO also has a role in preventing the misincorporation of FeMo‐cofactor into FeFe protein could not be evaluated. Nevertheless, this appears possible because *Rhodobacter capsulatus*, which encodes both Mo‐dependent and Fe‐only nitrogenases, but not V‐dependent nitrogenase, exhibit the same growth and catalytic activities associated with the inactivation of its *A. vinelandii* AnfO counterpart (Sicking et al., 2005). Based on the results reported here it is predicted that the phenotype of an AnfO‐deficient *R. capsulatus* would be rescued by inactivation of *nifEN*. However, exhaustive bioinformatic analyses have revealed that not all diazotrophic organisms that produce an Fe‐only nitrogenase also produce an AnfO (Addo & Dos Santos, 2020), indicating that the intrinsic structure of certain Fe‐only nitrogenases might prevent the misincorporation of an incorrect catalytic cofactor without the participation of AnfO or that other mechanisms exist to prevent incorrect cofactor insertion.

Why is there a physiological mechanism to prevent misincorporation of FeV‐cofactor and, perhaps, FeMo‐cofactor, into FeFe protein? One possibility is that such a mechanism could ensure maximum capacity for nitrogen fixation under conditions where the availability of V to sustain diazotrophic growth starts to become limiting. Namely, as cells transition from utilizing V‐dependent nitrogenase to a requirement for Fe‐only nitrogenase AnfO could ensure the fidelity of the initially produced FeFe protein by preventing the non‐productive misincorporation of FeV‐cofactor.

The present work reconciles the apparent discrepancies concerning the requirement of AnfO to sustain Fe‐only nitrogenase dependent diazotrophic growth in the native host *A. vinelandii*, but not in the heterologous bacterial host, *E. coli*, used in genetic reconstruction experiments. Such reconciliation was important from the perspective of experimental design in efforts to transfer a capacity for nitrogen fixation to model eukaryotic organisms. Namely, it was not previously established if the lack of a requirement for AnfO in genetic reconstruction experiments was the result of an ability of the heterologous host to replace AnfO function by using some other bacterial gene product, which would not necessarily be present in eukaryotic organisms. The present work excludes this possibility and demonstrates that preservation of AnfO function will not be necessary either to form or optimize an active Fe‐only nitrogenase in eukaryotic systems. In summary, a physiological role for AnfO involves the preservation of the fidelity of FeFe protein maturation by preventing misincorporation of FeV‐cofactor. Although the biochemical mechanism for AnfO function remains to be explored, two non‐exclusive possibilities are that AnfO transiently interacts with FeFe protein during its maturation resulting in a conformation that favors FeFe‐cofactor insertion and that AnfO sequesters FeV‐cofactor to prevent its misincorporation into FeFe protein during the maturation process. Phylogenetic comparisons of AnfO primary structures from a variety of diazotrophs that encode an Fe‐only nitrogenase (Figure S4) do not provide specific insights to indicate a probable mechanism for AnfO function. However, these phylogenetic comparisons do reveal that AnfO contains two conserved domains separated by a non‐conserved linker domain, indicating that it could be a modular protein having domains with distinct functions.

## 4 EXPERIMENTAL PROCEDURES

### 4.1 Strains used in this work

Details of the strains used in this study are listed in Table S1. All strains are derivatives of DJ1254, an *A. vinelandii* tungsten‐tolerant variant for which the structural genes encoding the MoFe protein are deleted (Yang et al., 2021). Where indicated, a Strep‐tag‐encoding sequence was inserted at the C‐terminal of *anfD* enabling one‐step‐affinity purification of FeFe protein. Strains DJ2621 and DJ2622 carry the *anfO* gene placed under control of the arabinose regulatory elements as previously described for other similar *A. vinelandii* genomic constructs (Dos Santos et al., 2007). For obtaining the pseudo‐revertant strains DJ2411 and DJ2490, dilutions of DJ2290 were plated on Burk’s modified nitrogen‐free medium plates lacking a Mo supplement and incubated for several days. Mutations within *vnfE* were determined by sequencing the complete *vnfENX* region of DJ2411 and DJ2490 (Genomics Sequencing Center [GSC], Fralin Life Sciences Institute, Virginia Tech). Deletion of the *vnfEN* genes to construct DJ2480 was performed as previously described (Pérez‐González et al., 2021).

### 4.2 Bacterial growth

*A. vinelandii* cells were grown at 30°C in Burk’s modified nitrogen‐free medium plates (Strandberg & Wilson, 1968) without the addition of molybdate. For non‐diazotrophic conditions, 13 mM ammonium acetate was added to the media as the nitrogen source. When indicated, sodium metavanadate (NaVO_3_) (Sigma‐Aldrich, St. Louis, USA) or sodium tungstate (Na_2_WO_4_) (Acros Organic, Geel, Belgium) was added to the media at concentrations indicated in the main text and/or figure. Where indicated, arabinose (Spectrum Chemical Mfg. Corp., Gardena, CA, USA) was added to the media at a final concentration of 3 g/L. For plates shown in Figure S1, Agarose (VWR, Randor, PA, USA) was used instead of Difco Agar (BD, NJ, USA) as the solidifying agent and salts used for media preparation were passed over a Chelex 100 resin (Biorad, Hercules, CA, USA) to reduce trace levels of V present in the growth media. Iron (II) sulfate heptahydrate (Fisher Scientific, Waltham, MA, USA) was added to the Chelex‐treated (18 μM final concentration) salts as the Fe source, and sodium metavanadate (5 μM final concentration) was added as the V source where indicated.

For large‐scale cultures, *A. vinelandii* cells were grown in a 150 L custom‐built fermenter (W.B.Moore, Inc., Easton, PA) at 30°C in a modified Burk’s medium containing 1 mM urea as the nitrogen source as previously described (Pérez‐González et al., 2021). Where indicated, 5 μM sodium metavanadate was added to the media as the V source. Cells were grown overnight and harvested approximately 8 to 10 h after exhaustion of fixed nitrogen.

### 4.3 Protein purification and analysis

Strep‐tagged FeFe proteins were purified following previously described procedures (Jiménez‐Vicente et al., 2018) using Strep‐Tactin columns (IBA Lifesciences, Göttingen, Germany). Fe‐protein‐3 was purified as previously described (Pérez‐González et al., 2021). Protein purity was assessed by SDS‐PAGE analysis. Protein concentrations were determined by the BCA method (BCA protein assay kit, Sigma‐Aldrich). The metal content (Fe, Mo, and V) was determined by inductively coupled plasma mass spectrometry (ICP‐MS) (Metal Analysis Service, Virginia Tech).

### 4.4 Substrate reduction assays

Substrate reduction assays were done using sealed 9.4 ml serum vials as previously described (Harris, Lukoyanov, et al., 2018). Vials contained 1 ml of the assay buffer which consisted of an ATP regeneration mixture (6.7 mM MgCl2, 30 mM phosphocreatine, 5 mM ATP, 0.2 mg/ml creatine phosphokinase) and 10 mM sodium dithionite in 100 mM MOPS buffer at pH 7.0. Solutions were made anaerobic and headspace gasses in the vials were adjusted to the desired partial pressures of the gaseous substrates (N_2_, C_2_H_2_, or CO) as indicated. Any remaining space was filled with Argon. Fe protein‐3 was added at saturation conditions to each assay vial and after that, those were ventilated to atmospheric pressure. Reactions were initiated by the addition of 0.1 mg FeFe protein. Reactions were incubated with agitation at 30°C for 8 min and quenched by the addition of 300 μl of 400 mM EDTA (pH 8.0). The products (NH_3_, H_2_, C_2_H_4_, C_2_H_6_, and CH_4_) from the different assays were quantified according to published methods (Benton et al., 2001; Corbin, 1984; Kim et al., 1995; Yang et al., 2011).

### 4.5 FeV‐cofactor isolation

FeV‐cofactor was isolated from pure FeFe protein following procedures previously described for FeMo‐cofactor (Srisantitham et al., 2021). Metal content of the isolated cofactor (Fe and V) was determined by inductively coupled plasma mass spectrometry (ICP‐MS) (Metal Analysis Service, Virginia Tech).

## CONFLICT OF INTEREST

The authors declare that there is no conflict of interest.

## AUTHOR CONTRIBUTIONS

*Conception and design of the study*: Ana Pérez‐González, Emilio Jimenez‐Vicente, Dennis R. Dean. *Acquisition of the data*: Ana Pérez‐González, Emilio Jimenez‐Vicente, Alvaro Salinero‐Lanzarote, Derek F. Harris. *Analysis and interpretation of the data*: Ana Pérez‐González, Emilio Jimenez‐Vicente, Alvaro Salinero‐Lanzarote, Derek F. Harris, Lance C. Seefeldt, Dennis R. Dean. *Writing of the manuscript*: Ana Pérez‐González, Dennis R. Dean.

## Supporting information


Supinfo

## Data Availability

The data that support the findings presented in this study are available within the article and its supplementary information.
