# Supplementary material for: AnfO controls fidelity of nitrogenase FeFe protein maturation by preventing misincorporation of FeV‐cofactor
Source: Mol Microbiol. 2022 Mar 9;117(5):1080–8. doi: 10.1111/mmi.14890 (PMC9310841; doi:10.1111/mmi.14890)
Supplement: Supplementary file 1 — Supinfo [file MMI-117-1080-s001.pdf]

# SUPPLEMENTARY INFORMATION

# AnfO controls fidelity of nitrogenase FeFe protein maturation by preventing misincorporation of FeV-cofactor

Ana Pérez-González[a], Emilio Jimenez-Vicente[a], Alvaro Salinero-Lanzarote[b], Derek F. Harris[c], Lance C. Seefeldt[c] and Dennis R. Dean[a]#

[a] Department of Biochemistry, Virginia Tech, Blacksburg, Virginia 24061, USA

[b] Centro de Biotecnología y Genómica de Plantas, Universidad Politécnica de Madrid, Instituto Nacional de Investigación y Tecnología Agraria y Alimentaria, Pozuelo de Alarcón, 28223 Madrid, Spain

[c] Department of Chemistry and Biochemistry, Utah State University, Logan, UT 84322, USA

#Address correspondence to Dennis R. Dean, deandr@vt.edu.

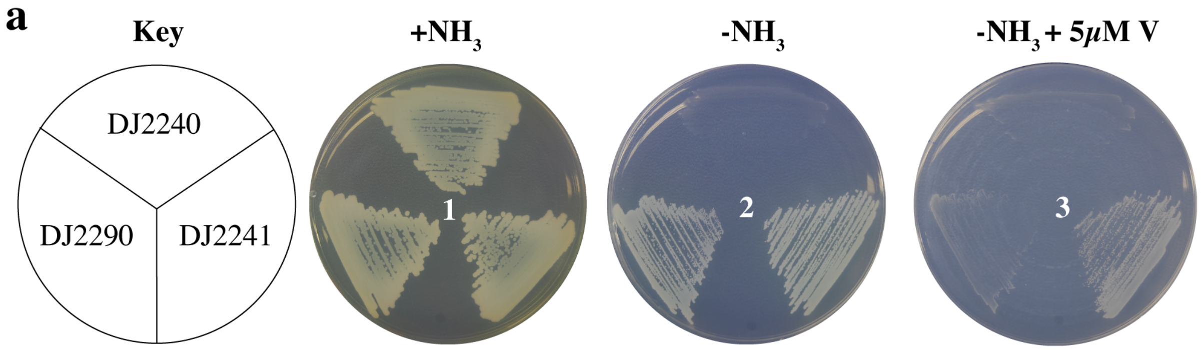


**b**

| Strain | Relevant Genes Inactivated | | |
|---|---|---|---|
| | ***Nif*** | ***Vnf*** | ***Anf*** |
| DJ2240 | *nifDK* | *vnfDGK* | *anfDGK* |
| DJ2241 | *nifDK* | *vnfDGK* | *none* |
| DJ2290 | *nifDK* | *vnfDGK* | *anfO* |

**Figure S1. Depletion of traces of vanadium in solid media reverses the null diazotrophic growth phenotype originally observed for DJ2290.** (a) Strains expressing FeFe nitrogenase were cultured on Burk's medium using agarose as the solidifying agent; metal ions contained in the salts used for media preparation were previously chelated using a Chelex 100 resin with the subsequent supplementation of Fe and V. Strains were cultured on plates containing a fixed nitrogen source (+$NH_3$) (plate 1) or under diazotrophic conditions (-$NH_3$) (plates 2 and 3) with or without addition of V, as indicated. Strains were cultured for 5 days. (b) Relevant genes inactivated in each strain. Refer to Table S1 for a complete genotypic description.

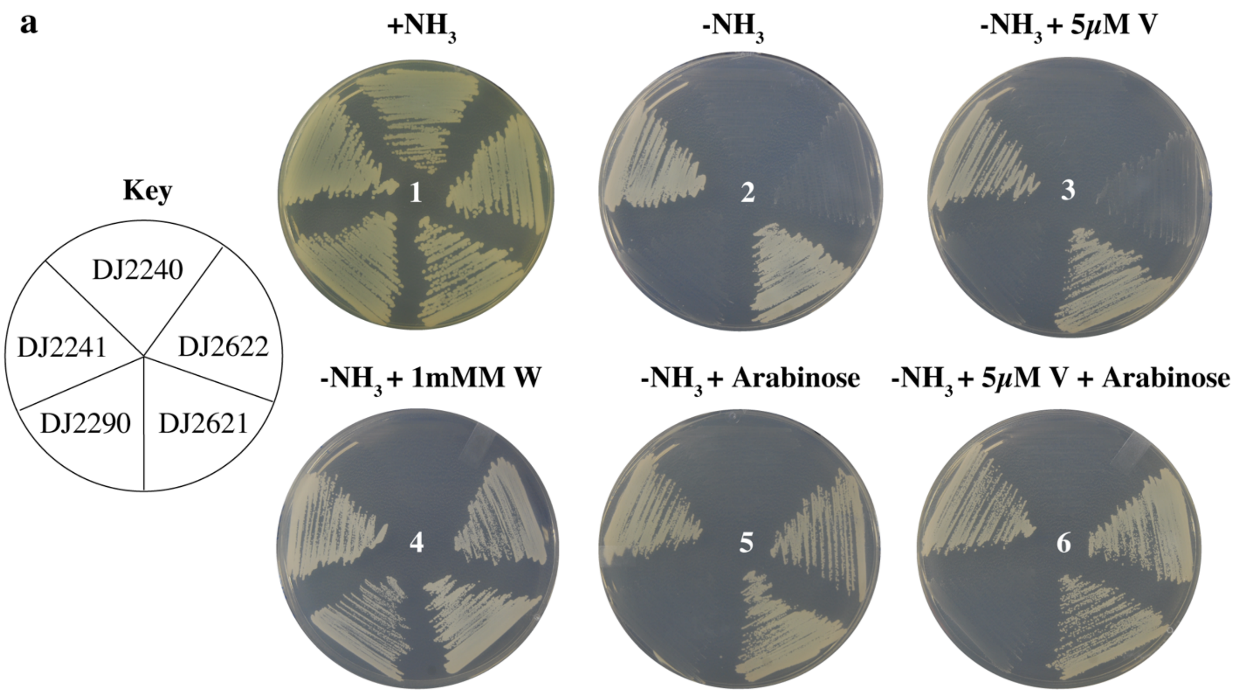


**b**

| Strain | Relevant Genes Inactivated | | | Arabinose induction of *anfO* expression |
|---|---|---|---|---|
| | ***Nif*** | ***Vnf*** | ***Anf*** | |
| DJ2240 | *nifDK* | *vnfDGK* | *anfDGK* | No |
| DJ2241 | *nifDK* | *vnfDGK* | *none* | No |
| DJ2290 | *nifDK* | *vnfDGK* | *anfO* | No |
| DJ2621 | *nifDK* | *vnfDGK vnfEN* | *none* | Yes |
| DJ2622 | *nifDK* | *vnfDGK vnfE* | *anfO* | Yes |

**Figure S2. Arabinose controlled expression of *anfO* reverses the null diazotrophic growth associated with deletion of the endogenous anfO.** (a) Strains expressing FeFe nitrogenase were cultured on Burk's medium agar plates containing a fixed nitrogen source (+$NH_3$) (plate 1) or under different diazotrophic growth conditions (-$NH_3$) (plates 2-5). Arabinose and V were added to the growth media as indicated for each condition. Strains were cultured on agar plates for 5 days. (b) Relevant genes inactivated in each strain. Refer to Table S1 for a complete genotypic description.  The important observation is that arabinose induced expression of *anfO* reverses the null diazotrophic growth phenotype associated with deletion of endogenous *anfO* whether V is added to the growth media.

**a**

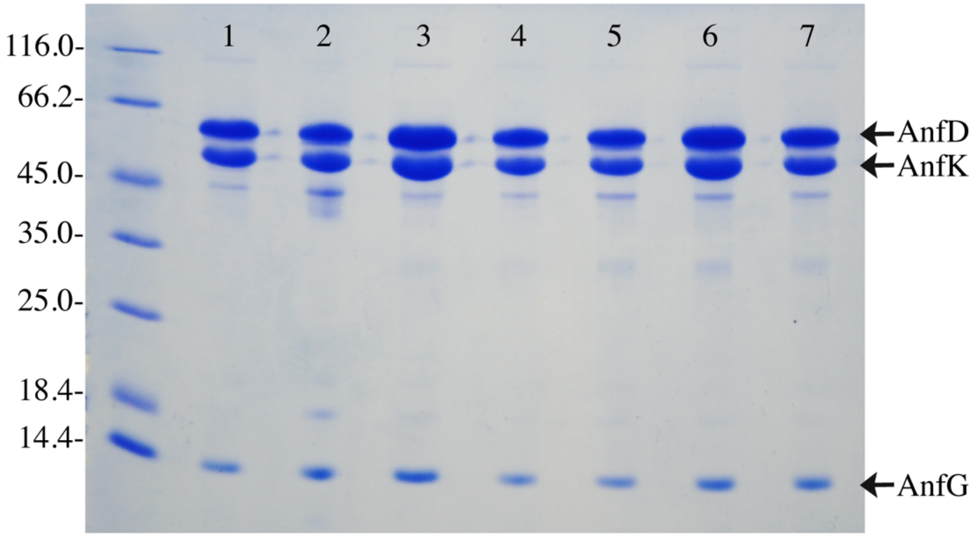


**b**

| Lane | Strain | Relevant Genes Inactivated | V added to the media |
|---|---|---|---|
| **1** | DJ2241 | *nifDK, vnfDGK* | *none* |
| **2** | DJ2290 | *nifDK, vnfDGK, anfO* | *none* |
| **3** | DJ2241 | *nifDK, vnfDGK* | 5μM V |
| **4** | DJ2290 | *nifDK, vnfDGK, anfO* | 5μM V |
| **5** | DJ2381 | *nifDK, vnfDGK, vnfEN* | 5μM V |
| **6** | DJ2411 | *nifDK, vnfDGK, vnfE, anfO* | 5μM V |
| **7** | DJ2480 | *nifDK, vnfDGK, vnfEN, anfO* | 5μM V |

**Figure S3. SDS-PAGE of FeFe protein purified from different strains and growth conditions.** (a) DJ2241 (wild type, lane 1 and 3); DJ2290 (inactivated for AnfO, lane 2 and 4); DJ2381 (inactivated for VnfEN, lane 5); DJ2411 (pseudo-revertant, inactivated for VnfE and AnfO, lane 6), DJ2480 (inactivated for VnfEN and AnfO, lane 7) (b) Relevant genes inactivated in each strain and growth conditions of the cells. Refer to Table S1 for a complete genotypic description. V=sodium metavanadate ($VO_3^{-1}$). “Wild-type” refers to the strain having the Anf-associated components intact. Standards located to the left of panel A indicate molecular weights in kDa. Note that the δ subunit (AnfG) is associated with the FeFe protein in all the samples analyzed.

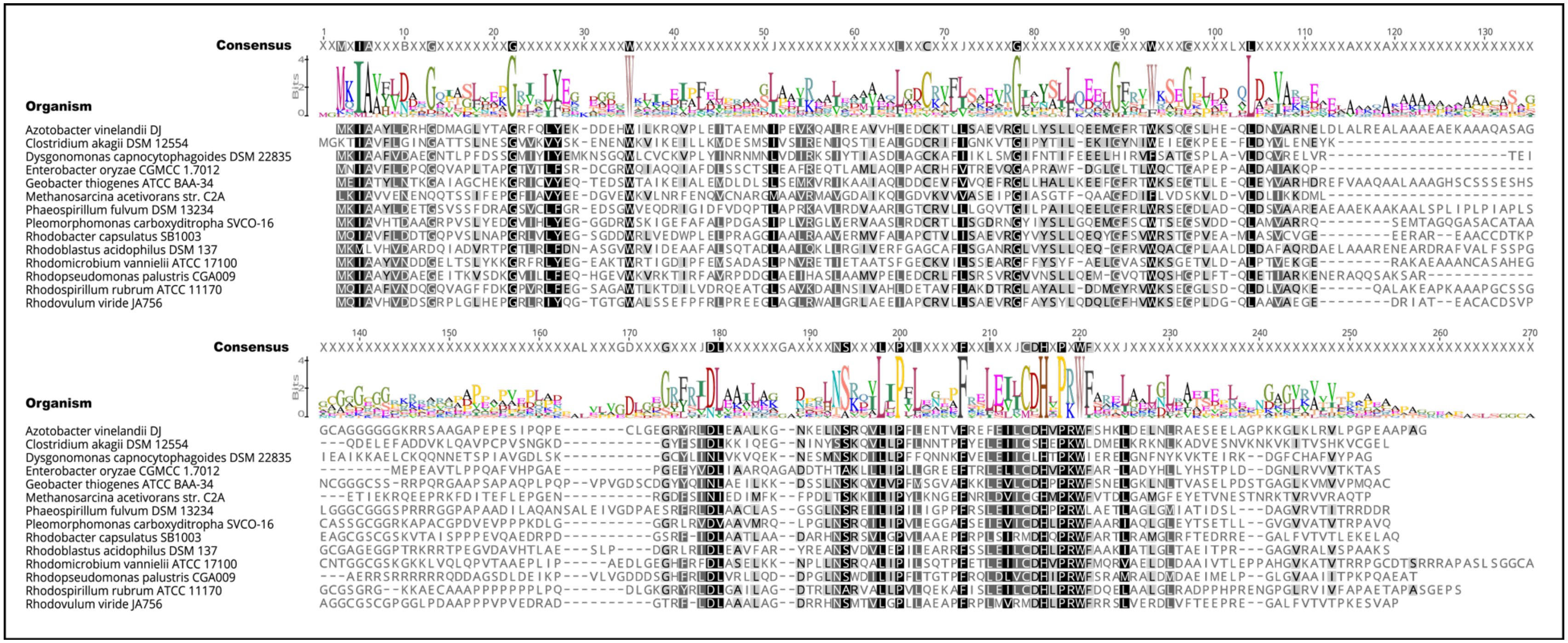


**Figure S4. Phylogenetic comparison of AnfO primary structures from a variety of diazotrophs that encode an Fe-only nitrogenase.** The AnfO primary structures of fourteen diazotrophs that encode an Fe-only nitrogenase were selected for a phylogenetic comparison. These organisms are representatives of different phylogenetic groups and respiratory requirements (for detailed information, see reference 33). This comparison revealed two conserved domains in AnfO separated by a non-conserved linker domain, indicating that AnfO could be a modular protein having domains with distinct functions. Amino acid sequences alignment was performed with Geneious 10.0.9 software (score matrix: Blosum62). Black squares indicate 100% similarity between the sequences; dark grey indicates 80-100% similarity; light grey indicates 60-80% similarity; white indicates less than 60% similarity.

**Table S1. List of *Azotobacter vinalendii* strains used in this work**.

The list includes the detailed genotypes for each strain used in this work. S-TAG: Strep-tag; km: kanamycin resistance cartridge; sm: spectinomycin resistance cartridge; Para: arabinose promoter. For strains DJ2621 and DJ2622 the *anfO* gene whose expression is under control of the *E. coli* arabinose regulatory elements was incorporated into the *scr*-region of the *A. vinelandii* genome as previously described[27].

| Strain | Genotype |
|---|---|
| DJ2240 | $\Delta$*nifDK*, $\Delta$*vnfDGK*::$sm^R$, $\Delta$*anfDGK*::$km^R$ |
| DJ2241 | $\Delta$*nifDK*, $\Delta$*vnfDGK*::$sm^R$, *anfD* $^{S\text{-}TAG}$ |
| DJ2290 | $\Delta$*nifDK*, $\Delta$*vnfDGK*::$sm^R$, $\Delta$*anfO, anfD* $^{S\text{-}TAG}$ |
| DJ2381 | $\Delta$*nifDK*, $\Delta$*vnfDGK*::$sm^R$, $\Delta$*vnfEN*, *anfD* $^{S\text{-}TAG}$ |
| DJ2411 | $\Delta$*nifDK*, $\Delta$*vnfDGK*::$sm^R$, $\Delta$*anfO*, $\Delta$*vnfE* $^{nt755}$, *anfD* $^{S\text{-}TAG}$ (*) |
| DJ2490 | $\Delta$*nifDK*, $\Delta$*vnfDGK*::$sm^R$, $\Delta$*anfO*, $\Delta$*vnfE* $^{nt827}$, *anfD* $^{S\text{-}TAG}$ (**) |
| DJ2480 | $\Delta$*nifDK*, $\Delta$*vnfDGK*::$sm^R$, $\Delta$*anfO*, $\Delta$*vnfEN, anfD* $^{S\text{-}TAG}$ |
| DJ2621 | $\Delta$*nifDK*, $\Delta$*vnfDGK*::$sm^R$, *Para::anfO*, *anfD* $^{S\text{-}TAG}$ |
| DJ2622 | $\Delta$*nifDK*, $\Delta$*vnfDGK*::$sm^R$, $\Delta$*anfO, Para::anfO*, *anfD* $^{S\text{-}TAG}$ |

Relevant residues removed as a result of gene deletions are indicated as follows:

- $\Delta$*nifDK* – NifD residue 103 to NifK residue 308 are deleted.
- $\Delta$*vnfDGK*::$sm^R$ - VnfD residue 271 and VnfK residue 202 are deleted and a streptomycin resistance cartridge is placed in exchange.
- $\Delta$*anfDGK* - AnfD residue 204 to AnfK residue 148 are deleted and a kanamycin resistance cartridge is placed in exchange.
- $\Delta$*anfO* - AnfO residue 179 to 224 are deleted.
- $\Delta$*vnfEN* - VnfE residue 67 to VnfN residue 414 are deleted.

(*) DJ2411 is a pseudo-revertant strain that contains a single bp frame shift "A" insertion after *vnfE* nucleotide 755.

(**) DJ2490 is a pseudo-revertant strain that carries a one bp frame shift "G" deletion of *vnfE* nucleotide 827
